# Malignant Melanoma in African–Americans: A Population-Based Clinical Outcomes Study Involving 1106 African–American Patients from the Surveillance, Epidemiology, and End Result (SEER) Database (1988–2011)

**DOI:** 10.1097/MD.0000000000006258

**Published:** 2017-04-14

**Authors:** Krishnaraj Mahendraraj, Komal Sidhu, Christine S.M. Lau, Georgia J. McRoy, Ronald S. Chamberlain, Franz O. Smith

**Affiliations:** a Department of Surgery, Saint Barnabas Medical Center, Livingston, NJ; b Saint George's University School of Medicine, Grenada, West Indies; c Department of Surgery, New Jersey Medical School, Rutgers University, Newark, NJ; d Department of Surgery, Banner MD Anderson Cancer Center, Gilbert, AZ.

**Keywords:** cutaneous melanoma, malignant melanoma, melanoma, SEER, skin cancer

## Abstract

Malignant melanoma accounts for 75% of all skin cancer deaths and is potentially curable if identified early. Although melanoma is rare in African–Americans (AA), it is associated with a worse prognosis than in Caucasians. This study examines the demographic, pathologic, and clinical factors impacting AA melanoma outcomes.

Data for 1106 AA and 212,721 Caucasian cutaneous melanoma patients were abstracted from the Surveillance, Epidemiology, and End Result (SEER) database (1988–2011). Data were grouped on the basis of histological subtypes: “Superficial Spreading” (SS), “Nodular” (NM), “Lentigo Maligna” (LM), “Acral Lentiginous” (AL), and “Not otherwise specified” (NOS).

Cutaneous malignant melanoma occurs most commonly in the sixth and seventh decade of life. Caucasian patients presented most commonly with trunk melanomas (34.5%), while lower extremity melanomas were more common in AAs (56.1%), *P* < 0.001. AAs presented with deeper tumors, more advanced stage of disease, and higher rates of ulceration and lymph node positivity than Caucasians. Cancer-specific mortality was significantly higher, while 5-year cancer-specific survival was significantly lower among AAs for NM and AL subtypes. Multivariate analysis identified male gender, regional and distant stage, NM and AL subtypes as independently associated with increased mortality among both ethnic groups.

AAs present most often with AL melanoma on the lower extremities, and with deeper and more advanced stage lesions. AAs have higher cancer-specific mortality for NM and LM than Caucasians. Melanoma education for AA patients and health care providers is needed to increase disease awareness, facilitate early detection, and promote access to effective treatment.

## 1 Introduction

According to the National Cancer Institute, cutaneous melanoma represents almost 5% of all new diagnosed cancer cases, with a reported mortality of approximately 2%, making it the deadliest form of skin cancer.^[1]^ The incidence of cutaneous melanoma has been steadily increasing over the last 10 years, and it is estimated that almost 1 million people are currently living with a melanoma diagnosis in the United States.^[1]^ Invasive cutaneous melanoma is the fifth most common cancer diagnosis among men and the seventh most common among women.^[1]^ Melanoma is far more common among Caucasians than African–Americans (AA), with incidence rates of 33.0 per 100,000 men and 20.2 per 100,000 women among Caucasians compared with 1.2 per 100,000 men and 1.0 per 100,000 women among AAs.^[1]^ Exposure to ultraviolet (UV) light is believed to be the most significant risk factor for developing cutaneous melanoma, based primarily on the observations that the incidence of melanoma is highest in populations with more direct sunlight and those living closer to the equator.^[2–6]^ Additional risk factors for melanoma in developed nations include the use of tanning beds and sunburns occurred during tanning among adolescents.^[2]^ The lower incidence rate of melanoma among dark skinned individuals is likely attributable to the protective effects of melanin.^[7]^

Although AAs have a significantly lower risk of melanoma than Caucasians, ethnic disparities with regard to histologic subtypes, anatomic distribution, stage at diagnosis, and survival have been well documented.^[6,8–13]^ In 1976, Reed ^[14]^ were the first to report the predominance of AL melanoma among AAs. A retrospective study of 1413 histologically confirmed cases of AL over a 19-year period (1986–2005) reported lower survival rates for AL melanoma among ethnic minorities including AAs, Hispanics, Asians, and Pacific Islanders than Caucasians.^[10]^ Previous studies have also demonstrated that ethnic minorities presented with more advanced disease, had thicker melanomas, as well as lower melanoma-specific survival than Caucasians in a retrospective study involving 288,741 cases of invasive melanoma over 7-year period (1999–2006).^[15]^ Of note, the AA sample size in each of these studies was quite low, and to date, there are no large-scale studies specifically analyzing melanoma among AAs, and as such, the explanation for the disparities in clinical outcomes among AA patients is poorly understood.^[10,15]^

This study sought to examine a large cohort of AA and Caucasian melanoma patients from the Surveillance, Epidemiology, and End Result (SEER) database, in an effort to identify demographic, clinical, and treatment strategies that impact clinical outcomes and survival.

## 2 Methods

Data for the current study were extracted from the SEER database provided by the National Cancer Institute between 1988 and 2011. SEER Stat software version 8.0.4 (National Institutes of Health (NIH) - National Cancer Institute, USA) was utilized to extract data from 18 SEER registries (Alaska Native Tumor Registry, Arizona Indians, Cherokee Nation, Connecticut, Detroit, Georgia Center for Cancer Statistics, Greater Bay Area Cancer Registry, Greater California, Hawaii, Iowa, Kentucky, Los Angeles, Louisiana, New Jersey, New Mexico, Seattle-Puget Sound, and Utah). Two hundred sixty-two thousand three hundred ninety-four cases of cutaneous melanoma were identified from the SEER database. Patients of Caucasian or AA race (213,827 patients) with cutaneous melanoma were identified and exported to IBM SPSSv20.2 (Armonk, NY). Five subgroups of melanoma were created for analysis using the SEER International Classification of Disease for Oncology (ICD-O-3) codes based on histological subtypes: “Superficial Spreading (SS)” (8743), “Nodular Melanoma (NM)” (8721), “Lentigo Maligna (LM)” (8742), “Acral Lentiginous (AL)” (8744), and “Malignant Melanoma, (NOS)” (8720). Demographic and clinical data extracted included age, gender, ethnicity, geographic region, tumor histology, site, depth, stage, grade, lymph node status, presence of ulceration, and type of treatment (surgery, radiation, both, or unknown/no therapy) received. Only Caucasians and AAs were examined. Endpoints examined included cancer-specific mortality, overall survival, and cancer-specific 2- and 5- year survival. Categorical variables were compared using the Chi-square test, and continuous variables were compared using *S*tudent *t* test, and analysis of variance (ANOVA). Multivariate analysis using the “backward wald” method was performed to calculate odds ratio (OR) and determine independent factors affecting survival. Missing and unknown data were excluded from the multivariate analysis. Kaplan–Meier analysis was used to compare long-term actuarial survival between groups. Statistical significance was accepted at the level of *P* < 0.05. Approval to conduct this study was obtained from Saint Barnabas Medical Center, and given the retrospective nature of the study involving data from the SEER database with no patient identifiable information, patient consent was not required.

## 3 Results

### 3.1 Demographic data

A total of 262,394 cases of cutaneous melanoma were identified from the SEER database (1988–2011). One thousand one hundred and six AA patients and 212,721 Caucasians were used to form the current study cohort. SS was the most common melanoma subtype among Caucasians (33.4% vs 15.6% AAs), while AL was the predominant subtype among AAs (18.0% vs 0.91% Caucasians), *P* < 0.001 (Table 1). Cutaneous melanoma (SS, NM, LM, AL, and NOS) occurred most commonly in the sixth to seventh decade of life, with Caucasians presenting slightly younger than AAs (58.9 ± 17.12 vs 60.5 ± 18.16 years, *P* < 0.001). Age at presentation was lowest among SS melanoma patients (55.1 ± 16.59 years for Caucasians, 55.49 ± 17.71 years for AAs) and highest in LM melanoma patients (69.9 ± 12.57 years for Caucasians, 66.93 ± 12.28 years for AAs), *P* < 0.001.

**Table 1:**
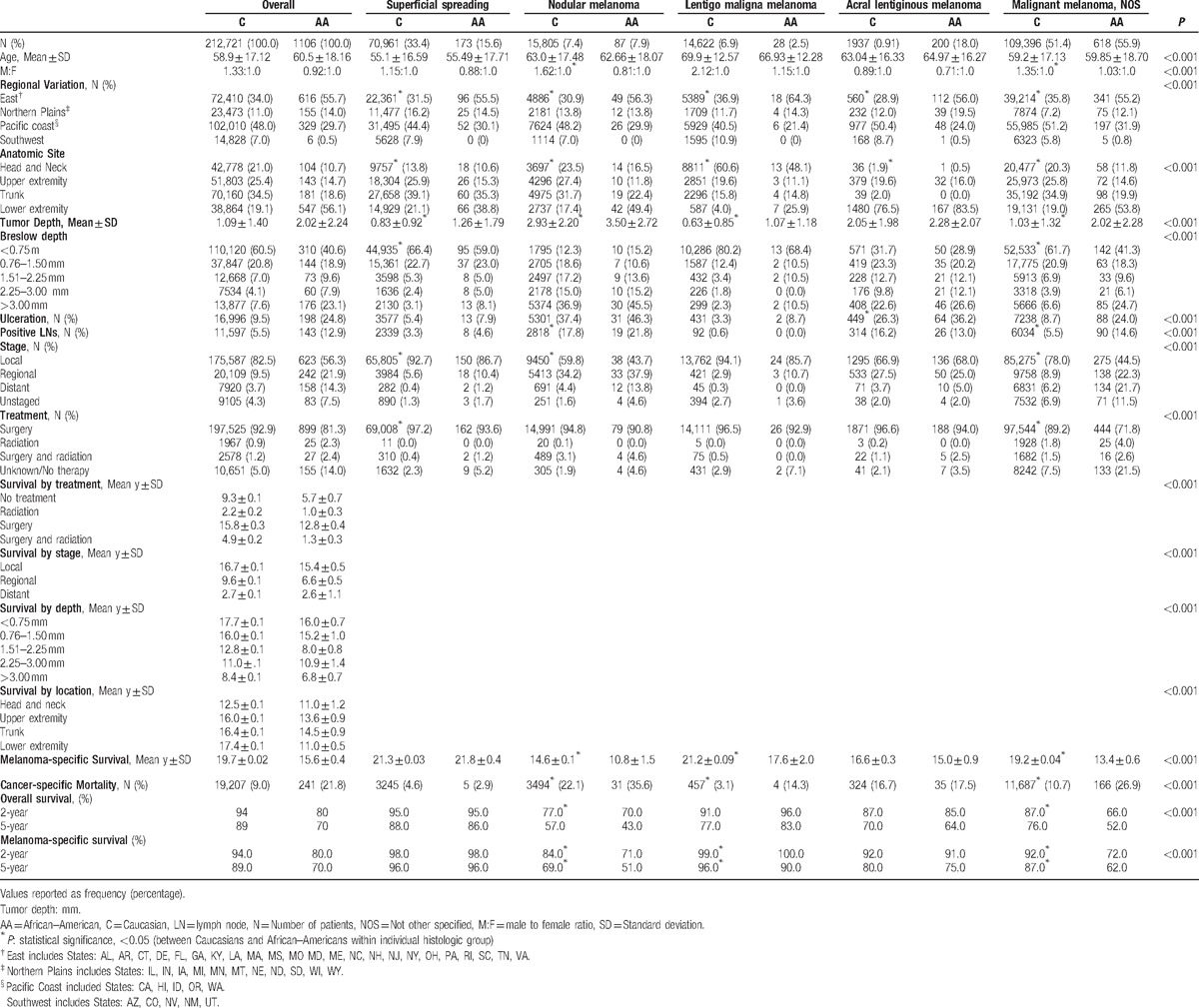
Demographic profiles, tumor characteristics, treatment, and survival outcomes of 1106 African–Americans and 212,721 Caucasian patients with malignant melanoma from the Surveillance, Epidemiology, and End Results (SEER) database, 1988–2011.

Significantly more Caucasian males and AA females had NM (male to female ratio of 1.62 : 1.0 and 0.81 : 1.0, respectively), *P* < 0.005. The highest incidence of melanoma diagnoses occurred on the East Coast among AAs (55.7%) and in the West Coast for Caucasians (48.0%), *P* < 0.001. This variation was consistent for all melanoma subtypes.

### 3.2 Tumor characteristics

Overall, more AAs (56.1%) presented with melanomas of the lower extremity than Caucasians (19.1%) for all tumor depths and stages, *P* < 0.001 (Table 2). Conversely, Caucasians most commonly presented with trunk melanomas (34.5%) for all tumor depths and stages, *P* < 0.001. The trunk was the most common disease site among Caucasians with both SS and NM (39.1% and 31.7%), while the lower extremity was the primary site for AAs with SS and NM (38.8% and 49.4%), *P* < 0.001. The majority of LM occurred on the head and neck in both Caucasians (60.6%) and AAs (48.1%), *P* < 0.001. AL occurred most commonly on the lower extremities for both Caucasians (76.5%) and AAs (83.5%), *P* = 0.04. A majority of ulcerated melanomas presented on the lower extremities among AAs (71.2%) and on the trunk among Caucasians (31.3%), *P* < 0.001.

**Table 2:**
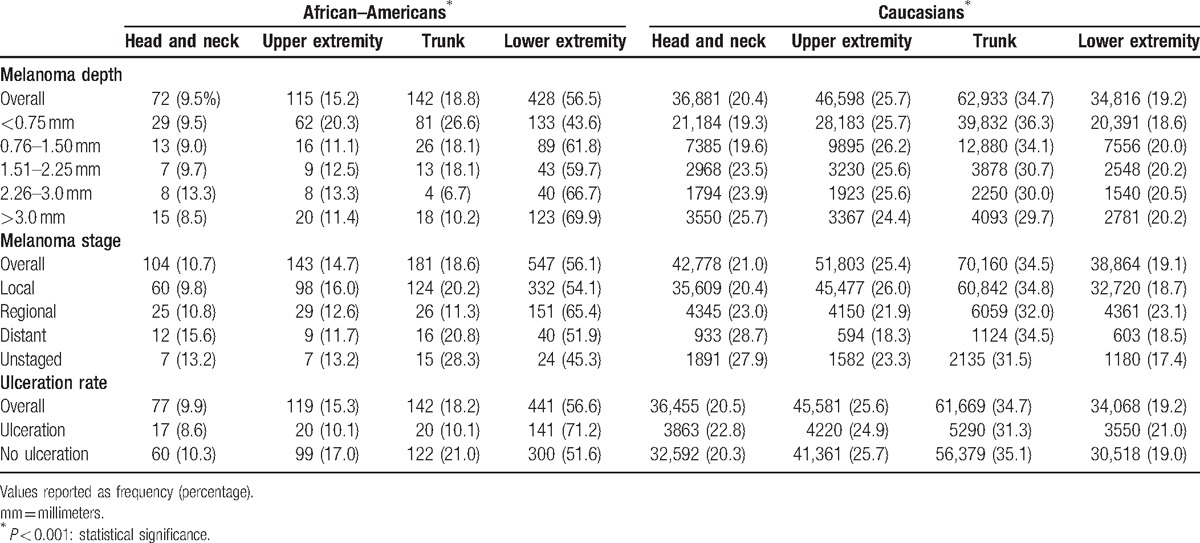
Melanoma depth, stage, and ulceration rates by location among 1106 African–Americans and 212,721 Caucasian patients with malignant melanoma from the Surveillance, Epidemiology, and End Results (SEER) database, 1988–2011.

Overall, AAs presented with deeper tumors than Caucasians for SS (1.26 vs 0.83 mm), NM (3.50 vs 2.93 mm), and LM (1.07 vs 0.63 mm) melanoma, respectively, *P* < 0.001. AL melanomas were also deeper in AAs (2.28 vs. 2.05 mm), although the difference did not reach statistical significance, *P* = 0.142. Among SS melanoma, 8.1% of AAs presented with tumors deeper than 3.00 mm compared to only 3.1% of Caucasians, *P* < 0.001.

AAs were significantly more likely to present with melanoma tumor ulceration than Caucasians for AL (36.2% vs 26.3%, *P* < 0.05), but not for the other subtypes. With regard to lymph node invasion, 21.8% of AAs with NM had lymph node positivity compared with 17.8% Caucasians, *P* = 0.01. Overall, AAs presented most commonly with melanomas of the lower limbs, deeper tumors, greater ulceration rates, higher rates of lymph node involvement, and with more advanced stage than Caucasians, *P* < 0.001.

When stratified by disease stage, Caucasians predominantly presented with localized disease in the SS subtype (92.7%), *P* < 0.001. On the contrary, AAs with SS melanomas had a significantly higher incidence of regionally advanced (10.4%) and distant disease (1.2%) than Caucasians (5.6% and 0.4%, respectively), *P* < 0.05. A similar pattern was observed for NM, where a majority of Caucasians presented with localized disease (59.8%), while AAs presented predominantly with regionally advanced (37.9%) and distant disease (13.8%) compared with Caucasians (34.2% and 4.4%, respectively), *P* < 0.001.

### 3.3 Treatment and outcomes

The majority of both ethnic groups were treated surgically (92.8%). A significantly higher proportion of Caucasians (92.9%) were treated surgically than AAs (81.3%), *P* < 0.001. More Caucasians than AAs underwent surgery for all stages of disease: localized (97.8% vs 96.0%), regional (95.7% vs 88.9%), and distant disease (74.1% vs 50.0%), *P* < 0.001 (Table 3). A greater proportion of Caucasians (97.2%) underwent surgical resection for SS melanoma than AAs (93.6%), *P* = 0.035. No significant treatment difference was noted among NM, LM, and AL between Caucasians and AAs.

**Table 3:**
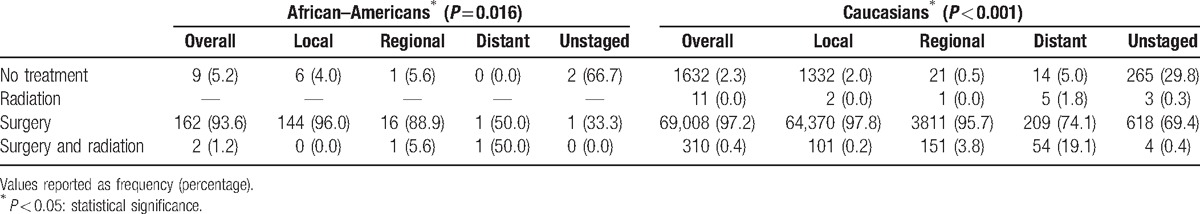
Melanoma stage by type of treatment received among 1106 African–Americans and 212,721 Caucasian patients with malignant melanoma from the Surveillance, Epidemiology, and End Results (SEER) database, 1988–2011.

**Figure 1:**
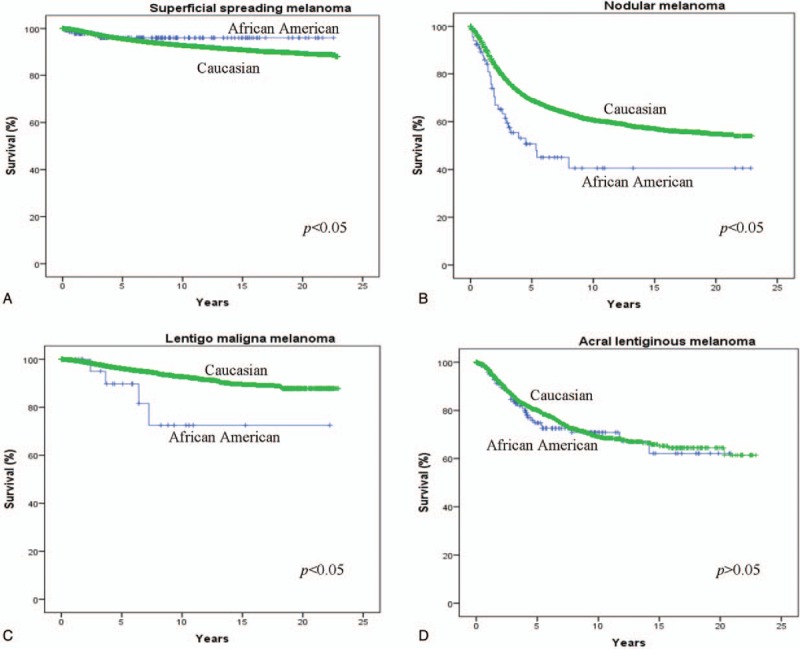
Kaplan–Meier actuarial survival among 1106 African–Americans and 212,721 Caucasians with (A) superficial spreading melanoma, (B) nodular melanoma, (C) Lentigo maligna melanoma, and (D) acral lentiginous melanoma from the Surveillance Epidemiology and End Result (SEER) Database (1988–2011).

There was no significant difference in overall mortality between ethnic groups for all melanoma subtypes. The mean melanoma-specific survival was lower among AAs than among Caucasians (10.8 ± 0.1 vs 14.6 ± 0.1 years, respectively, *P* < 0.001) for NM. Similarly, melanoma-specific survival was lower for AAs than Caucasians for LM (17.6 ± 2.0 vs 21.2 ± 0.09 years, respectively, *P* < 0.050). For NM and LM subtypes, melanoma-specific mortality was higher in AAs (NM; 35.6%, LM; 14.3%) than Caucasians (NM; 22.1%, LM; 3.1%), *P* < 0.005. Surgery was associated with prolonged survival among both ethnic groups; however, survival after surgery was significantly longer in Caucasians (15.8 years) than AAs (12.8 years), *P* < 0.001. Two-year melanoma-specific survival was highest for SS melanoma (98.0% in Caucasians and AAs both) and lowest for NM (84.0% in Caucasians and 71.0% in AAs). Similarly, 5-year survival was highest for SS subtype (96.0% in Caucasians and AAs both) and lowest for NM (69.0% for Caucasians and 51.0% in AAs). A significantly lower 5-year melanoma-specific survival was noted among AAs compared with Caucasians for certain melanoma subtypes, including NM (69.0% for Caucasians and 51.0% in AAs) and LM (96.0% for Caucasians and 90.0% in AAs), *P* < 0.05 (Fig. 1).

### 3.4 Multivariate analysis

On multivariate analysis of the overall melanoma cohort, male gender [OR 1.4; confidence interval (CI) = 1.3–1.5], AA ethnicity (OR 1.5; CI = 1.2–2.0), NM (OR 1.7; CI = 1.6–1.8), and AL (OR 1.8; CI = 1.6–2.2) histology, head and neck (OR 1.4; CI = 1.3–1.5), and trunk (OR 1.3; CI = 1.2–1.4) primary tumor site, tumor depth >0.75 mm (OR 3.3; CI = 3.1–3.6), regional (OR 3.8; CI = 3.5–4.1) and distant tumor stage (OR 7.5; CI = 6.3–8.9), ulceration (OR 1.1; CI = 1.0–1.2), and lymph node positivity (OR 1.2; CI = 1.1–1.3) were identified as risk factors independently associated with increased mortality, *P* < 0.001.

Among AA melanoma patients, factors independently associated with increased mortality included male gender (OR 1.5; CI = 1.1–2.2), regional (OR 4.3; CI = 2.5–7.3) and distant stage (OR 3.7; CI = 1.3–10.5), tumor depth >1.51 mm (OR 4.0; CI = 2.0–7.8), NM (OR 2.6; CI = 1.2–5.9), and AL (OR 1.9; CI = 1.1–3.3), *P* < 0.05.

Among Caucasian melanoma patients, male gender (OR 1.5; CI = 1.5–1.6), northern plains (OR 1.2; CI = 1.1–1.2), tumor depth >0.76 mm (OR 2.1; CI = 2.0–2.2), regional (OR 3.0; CI = 2.8–3.2), distant disease (OR 4.7; CI = 4.0–5.4), NM (OR 1.5; CI = 1.5–1.6), LM (OR 1.7; CI = 1.6–1.8), and AL (OR 1.9; CI = 1.7–2.1) were all independently associated with increased mortality, *P* < 0.001. Surgical resection conferred a significant survival advantage (OR 0.2; CI = 0.1–0.5), *P* < 0.001.

## 4 Discussion

Melanoma is a deadly form of skin cancer derived from pigment-producing melanocytes.^[1,16,17]^ The incidence of cutaneous melanoma has been steadily increasing over the last decade, representing almost 5% of all newly diagnosed cancer cases, and affecting approximately 1 million Americans.^[1,17]^ Fair skin and prolonged UV exposure from sunlight have been documented as major risk factors for developing cutaneous melanoma.^[16,17]^ Although melanoma is far more common among Caucasians, the prognosis for AAs diagnosed with melanoma is substantially worse.^[5]^ Cormier et al^[5]^ reported that the incidence of cutaneous melanoma was significantly lower in ethnic minority populations. Although all minority populations had worse prognosis than Caucasians, AAs showed the greatest ethnic discrepancy.^[5]^ AAs were 4 times more likely to present with advanced stage IV melanoma and 1.5 times more likely to die from melanoma than Caucasians.^[5]^

Cutaneous melanoma occurs most commonly in the sixth and seventh decade of life in both Caucasians and AAs. Although melanoma is more common among Caucasian females in the trunk and torso regions, over half of all melanomas among AA males involve the lower extremities. Numerous prior studies have reported similar results with AA melanomas occurring more often on nonsun-exposed skin, such as the palms and soles of the feet where there is less pigment and less melanin to protect the melanocytes from UV radiation.^[5,16,18]^

The most common form of melanoma in the AA population is acral lentiginous melanoma, whereas superficial spreading melanoma is the most common among Caucasians. This is consistent with previous studies, which report AL melanoma to be significantly more common among AAs than among Caucasians.^[5,10]^ This histological variation and clinical heterogeneity among melanomas represent an additional contributing factor to the poorer prognosis among AA melanoma patients. The atypical location of these lesions has also been reported to delay diagnosis specifically among plantar melanomas that typically present with significantly deeper tumors than nonplantar tumors (2.55 vs 1.22 mm).^[19,20]^ Franke et al^[19]^ attributed the poorer prognosis of AL to a delay in diagnosis, and reported an average of 4.8 years before patients sought medical attention, and a 7-month delay before receiving adequate surgical treatment. Shorter survival and higher mortality rates have also been reported with AL melanomas that are also more common among AAs.^[5]^ A retrospective study involving 1413 patients with AL melanoma demonstrated that the highest proportion of AL melanomas occurred among AAs (36%).^[10]^ AL melanoma is associated with significantly lower 5- and 10-year survival rates than all other cutaneous melanomas subtypes (80.3% and 67.5% vs 91.3% and 87.5%, *P* < 0.001).^[10]^ Moreover, AL melanoma is associated with a 12-fold increase in the risk of developing stage IV melanoma and an almost 2-fold increase in the risk of mortality in both Caucasians and AAs.^[5]^

Most melanomas present as localized disease; however, AA patients are far more likely to present with advanced disease and deeper tumors than Caucasians. AA melanoma patients also exhibit higher rates of regional and distant disease, and lower rates of localized disease than Caucasians, and are twice as likely to present with ulcerated lesions, metastatic disease, and lymph node involvement. In the current study, regional and distant disease, as well as tumor depth >1.51 mm were also associated with increased mortality for both Caucasian and AAs, which is consistent with prior reports.^[5,18]^

Surgical resection is the most common treatment modality for patients with localized cutaneous melanoma.^[21]^ In the current study, AAs were far less likely to receive surgical resection compared with Caucasians, even though surgical resection was associated with a significantly improved survival in all patient groups. A prior SEER study involving 151,154 patients with primary cutaneous melanoma reported that Caucasians were significantly more likely to receive appropriate surgical therapy than AAs (94.5% vs 86.6%, *P* < 0.05).^[13]^ Among those who received surgical treatment, AAs experienced significantly lower 5-year (66.8% vs 84.2%, *P* < 0.0001) and 10-year survival (55.4% vs 74.3%, *P* < 0.001) than Caucasians, possibly attributable to more advanced stage and increased rates of ulceration.^[13]^ In addition, it has been reported that AAs are less likely to receive adequate wide excision surgery (69.3% vs 77.7%) and more likely to receive local tumor excision (14.5% vs. 11.2%) than Caucasians, *P* < 0.001.^[22]^ Ethnic disparities and the suboptimal application of cancer-directed treatments, including surgery, radiation, and chemotherapy is not unique to melanoma and has been well documented for a wide variety of malignancies.^[13,23–25]^

In melanoma, advanced stage at presentation often leads to poor prognosis, and AA melanoma patients experience lower mean survival times as well as lower overall and cancer-specific 5-year survivals. A retrospective SEER study involving 49,772 melanoma patients demonstrated a significantly lower 5-year survival among AAs than Caucasians (72.2% vs 89.6%, *P* < 0.001).^[5]^

In addition to the obvious increased difficulty in diagnosing melanoma on the acral surfaces of the body, the more aggressive tumor biology, and more advanced stage at presentation, additional factors including lower socioeconomic status and limited access to health care services have also been implicated or potentially contribute to the disproportionate number of cancer deaths among AAs.^[5,18,26–28]^ Low socioeconomic status has been strongly associated with later stages at melanoma presentation and lower survival rates than higher socioeconomic groups.^[26–28]^ A population-based study involving 29,792 melanoma cases in California reported that the lowest socioeconomic status group experienced the steepest rise in the incidence of thick melanomas >4 mm.^[26]^ In a separate report by Chang et al,^[28]^ lower socioeconomic status was strongly associated with lower 5-year survival among early (83.2% vs 90.9%, *P* < 0.05) and late-stage melanoma patients (30.0% vs 45.5%, *P* > 0.05). Saraiya et al^[27]^ conducted a survey involving over 75,000 respondents, and reported lower rates of recent skin examinations among AAs than Caucasians in 1992 (5.8% vs 11.4%) and 2000 (6.2% vs 8.9%). Patients with higher education were also significantly more likely to have had a recent skin examination (14.5% of college graduates vs. 6.0% of high school graduates and 3.4% of individuals without a high school diploma, *P* < 0.001).^[27]^ The authors also reported that Caucasian adults >50 years of age (OR 1.57), high school education or more (OR 2.84), patients who had consistent health care (OR 2.18), and health insurance (OR 1.61) were more likely to receive recent skin examinations, *P* < 0.001.^[27]^

Understanding differences in the presentation between Caucasians and AAs is crucial to permit early diagnosis and treatment for this disease. Increased education and awareness of melanoma among minority populations may provide an opportunity for screening and early detection. Current public health education programs and screening are targeted primarily at fair skin Caucasians with prolonged UV sun exposure, and exclude AAs entirely.^[18]^ Efforts at educating physicians about the unique features of melanoma among AA patients are required to successfully provide screening for early detection among all ethnic backgrounds. Patients who had total body skin evaluations were 6 times more likely to have a melanoma detected than those who only received partial skin examinations.^[29]^

There are several limitations to this study that should be taken into account. First, the SEER database did not accurately code for important clinical factors such as socioeconomic status, access to appropriate medical facilities, method of diagnostic confirmation, and comorbidities, which may have had an influence on survival. Second, availability of screening programs and follow-up information was lacking. Data on surgical and radiation therapy utilized were available in the SEER database; however, information on surgical resection margins and chemotherapy received was not, and this limits the study's ability to comment on the impact of adjuvant or neoadjuvant therapy. Furthermore, the sample size in some groups (e.g., AAs with lentigo maligna melanoma) was small, making it difficult to draw conclusions. There may also be an element of selection bias in this data set, as SEER registries are more likely to sample from urban rather than rural areas. Despite these limitations, the SEER database contains data from 26% of the United States population, and these findings can be generalized to the overall population.

## 5 Conclusion

Despite the documented rise in the incidence of melanoma in recent decades, cutaneous melanoma remains a rare disease among AA patients. In contrast to Caucasians patients who present most often with superficial spreading melanoma, AAs most commonly present with acral lentiginous melanoma. Moreover, AAs typically have melanomas on the lower extremities, have tumors with greater depth and ulcer rates, and increased lymph node positive melanoma rates. Surgery is the preferred treatment and significantly prolongs survival in all affected patients; however, AAs experience significantly shorter survival and higher overall and melanoma-specific mortality. AAs experienced worse survival when stratified by tumor location, depth, stage, and treatment type than Caucasians. AAs with NM and LM melanoma have lower survival than Caucasians; however, AAs with SS melanoma was associated with slightly longer survival than Caucasians. Difficulty associated with diagnosing melanoma on acral surfaces, more aggressive tumor biology, more advanced depth and stage at presentation, as well as lower socioeconomic status and more limited access to health care services all contribute to the poor prognosis seen with AA melanoma patient population. Educating physicians about the unique features of melanoma among AA patients as well as increasing melanoma awareness among minority populations in crucial to improving screening and early detection rates that can assure appropriate and adequate treatment. Given the high mortality among AA melanoma patients, additional studies investigating the differences in tumor biology and genetic mechanisms between ethnic groups are required to precisely identify factors leading to lower survival rates among AA melanoma patients and identify optimal treatment for these patients.
